# Sesquiterpenoids from *Tussilago farfara* Flower Bud Extract for the Eco-Friendly Synthesis of Silver and Gold Nanoparticles Possessing Antibacterial and Anticancer Activities

**DOI:** 10.3390/nano9060819

**Published:** 2019-05-31

**Authors:** You Jeong Lee, Kwangho Song, Song-Hyun Cha, Seonho Cho, Yeong Shik Kim, Youmie Park

**Affiliations:** 1 College of Pharmacy and Inje Institute of Pharmaceutical Sciences and Research, Inje University, 197 Inje-ro, Gimhae, Gyeongnam 50834, Korea; ouoworld39@gmail.com; 2 College of Pharmacy and Natural Products Research Institute, Seoul National University, 1 Gwanak-ro, Gwanak-gu, Seoul 08826, Korea; siwcazb@snu.ac.kr (K.S.); kims@snu.ac.kr (Y.S.K.); 3 Department of Naval Architecture and Ocean Engineering, Seoul National University, 1 Gwanak-ro, Gwanak-gu, Seoul 08826, Korea; chasonghyun@snu.ac.kr (S.-H.C.); secho@snu.ac.kr (S.C.)

**Keywords:** silver nanoparticles, gold nanoparticles, *Tussilago farfara*, antibacterial activity, anticancer activity, sesquiterpenoids

## Abstract

Sesquiterpenoids from the flower bud extract of *Tussilago farfara* were effectively utilized as a reducing agent for eco-friendly synthesis of silver and gold nanoparticles. The silver and gold nanoparticles had a characteristic surface plasmon resonance at 416 nm and 538 nm, respectively. Microscopic images revealed that both nanoparticles were spherical, and their size was measured to be 13.57 ± 3.26 nm for the silver nanoparticles and 18.20 ± 4.11 nm for the gold nanoparticles. The crystal structure was determined to be face-centered cubic by X-ray diffraction. Colloidal stability of the nanoparticle solution was retained in a full medium, which was used in the cell culture experiment. The antibacterial activity result demonstrated that the silver nanoparticles showed better activity (two- to four-fold enhancement) than the extract alone on both Gram-positive and Gram-negative bacteria. Interestingly, the highest antibacterial activity was obtained against vancomycin-resistant Enterococci Van-A type *Enterococcus faecium*. Cytotoxicity on cancer cell lines confirmed that gold nanoparticles were more cytotoxic than silver nanoparticles. The highest cytotoxicity was observed on human pancreas ductal adenocarcinoma cells. Therefore, both nanoparticles synthesized with the sesquiterpenoids from *T. farfara* flower bud extract can be applicable as drug delivery vehicles of anticancer or antibacterial agents for future nanomedicine applications.

## 1. Introduction

Both silver nanoparticles (AgNPs) and gold nanoparticles (AuNPs) have attracted the attention of researchers owing to their various applications as antimicrobial agents, drug delivery vehicles for cancer and other disease treatments, chemical/biological sensors, and catalysts [1,2,3,4,5,6]. Silver (Ag) has been used since ancient times as an antimicrobial agent. It has been reported that AgNPs possess antibacterial activity against both Gram-positive and Gram-negative bacteria [7,8]. The high surface area-to-volume ratio and characteristic physicochemical properties contribute to the excellent antibacterial activity of AgNPs. Recently, the emergence of antibiotic resistance has been increasing due to multidrug-resistant (MDR) pathogens. Therefore, there is a need for the development of antibacterial agents with more efficacy, less toxicity, and more cost-effectiveness for the treatment of MDR infections. AgNPs can deliver decent platforms for this purpose. Factors affecting the antibacterial activity of AgNPs include size, shape, concentration, and dose [9]. AgNPs have been reported as antibacterial agents against the following MDR strains: vancomycin-resistant *Staphylococcus aureus*, methicillin-resistant *S. aureus*, ampicillin-resistant *Escherichia coli*, erythromycin-resistant *Streptococcus pyogenes*, and methicillin-resistant *S. epidermis* [9]. Surfactant- or polymer-modified AgNPs were effective against vancomycin-resistant *Enterococcus faecium* (VRE) when compared with unmodified AgNPs [10].

The antibacterial activity of AgNPs starts by the contact of AgNPs with the bacterial surface [4,11,12,13]. The accumulation of AgNPs on the surface of bacteria results in damage to the bacterial cell wall. This damage can induce morphological changes in the cell membrane, leading to changes in cell permeability and cell death. The acting mechanism was affected by the properties of AgNPs, such as shape, size, concentration and surface modification/functionalization [4,8,12,13]. It has been reported that small-sized AgNPs showed high antibacterial activity [11,13]. Interestingly, rod and triangular-shaped AgNPs displayed higher antibacterial activity than spherical ones [14,15,16,17]. One of smart strategies to combat bacterial resistance is a combination of AgNPs with antibiotics. Naqvi and co-workers reported that combination therapy against MDR bacteria was effective by a two- to seven-fold increase in activity [18]. Against MDR *Salmonella typhimurium* DT104, the combination of AgNPs with either tetracycline or neomycin was effective in a dose-dependent manner [19]. AuNPs have been exploited in the field of biomedicine due to their intrinsic features [20]. Specifically, AuNPs are applicable in photodynamic and photothermal therapy, X-ray imaging, drug delivery, and sensing [20]. AuNPs are very prevalent as delivery vehicles due to their high biocompatibility, low cytotoxicity, simple synthesis and facile surface coatings via covalent bonding or physical adsorption [21]. AuNPs can be modified with diverse ligands on their surface, such as anticancer agents, to apply as drug delivery vehicles [21]. The size and shape of AuNPs must be considered during synthetic steps, as cytotoxicity can be greatly influenced [22]. Sphere and rod shaped AuNPs were shown to be more cytotoxic than star, flower, and prism shaped ones [22]. Irrespective of cell types tested, smaller sized AuNPs were more cytotoxic than larger AuNPs [23]. Furthermore, surface functionalization also affects the cytotoxicity of AuNPs [24]. Cetyltrimethylammonium bromide-coated AuNPs were found to be more cytotoxic than AuNPs functionalized with polyethylene glycol and human serum albumin.

Both AgNPs and AuNPs can be synthesized by chemical, biological, and physical methods. Currently, eco-friendly synthesis (also known as green synthesis or sustainable synthesis) of these nanoparticles is an important issue for the protection of our global environment. With increasing global sustainability initiatives, plant extracts with diverse biological activities are considered significant natural resources for eco-friendly synthesis [25,26]. Advantages of eco-friendly synthesis using plant extracts include the following: (i) easily obtainable raw materials, (ii) cost efficiency, (iii) amenability to reaction scale-up, (iv) a facile reaction process, and finally (v) environmental friendliness [27]. Phytochemicals in plant extracts are primary or secondary metabolites of plants and generally possess effective biological activities that benefit human health. Furthermore, eco-friendly synthesis using plant extracts eliminates the use of toxic chemicals, such as sodium borohydride and hydrazine. Different parts of plants, such as the leaf, root, rhizome, flower, fruit, petal, aerial part, and whole plant, are being utilized for synthesis [27]. Diverse functional groups of phytochemicals are capable of processing a reduction reaction of Ag or Au salts to AgNPs or AuNPs, respectively. Most notably, the biological activities of the resulting nanoparticles can be reinforced due to the synergistic effects of combining both intrinsic activities from nanoparticles and plant extracts.

Both AgNPs and AuNPs synthesized with plant extracts have potential applications in cancer theranostics [27]. The anticancer activity of these green nanomaterials has been evaluated against diverse cancer cell lines, such as the human colorectal adenocarcinoma cell (HT-29), the human lung carcinoma cell (A549), the human hepatocellular carcinoma cell (HepG2), the human cervix adenocarcinoma cell (HeLa), the human breast adenocarcinoma cell (MCF-7), the human skin malignant melanoma cell (A375), the Mus musculus skin melanoma cell (B16F10), the human breast adenocarcinoma cell (MDA-MB-231), and the human colon adenocarcinoma cell (Colo 205) [27,28]. The mechanism of action of these green nanomaterials synthesized with plant extracts includes reactive oxygen species (ROS)-dependent apoptosis and caspase-mediated apoptosis in cancer cell lines [29,30].

*Tussilago farfara* (Asteraceae) is a perennial plant used in traditional medicine. Farfarae Flos, which is the flower bud of *T. farfara,* has been used in the treatment of asthmatic conditions, such as coughs and bronchitis. Tussilagone, a major active compound of *T. farfara,* inhibits inflammatory responses and improves survival in septic mice [31]. The flower buds of *T. farfara* show neuroprotective effects in ischemic stroke [32], inhibit enterovirus-induced cell injury [33], and demonstrate cytoprotective activity against glucose oxidase-induced oxidative stress [34]. Sesquiterpenoids from *T. farfara* inhibit nitric oxide production [35,36], show neuroprotective activity [37,38], and inhibit diacylglycerol acyltransferase activity [39]. Specifically, tussilagone suppresses colon cancer cell proliferation [40], and *T. farfara* augments apoptosis in human hepatocellular carcinoma cells [41]. These two reports suggest that *T. farfara* and tussilagone can be potential chemotherapeutics in cancer therapy.

**Figure 1:**
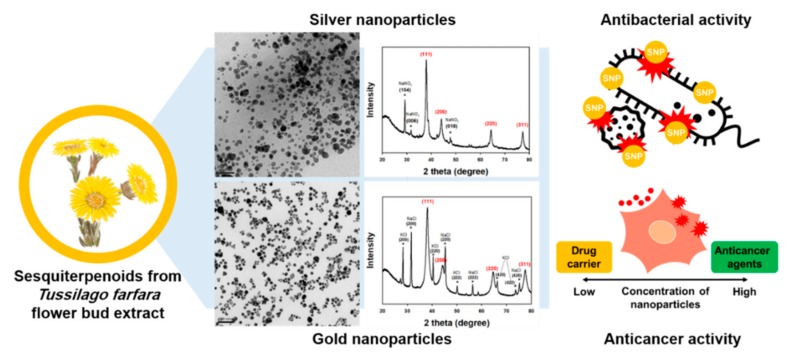
A schematic presentation of the current report.

In the current report, sesquiterpenoids from *T. farfara* flower bud extract were utilized as a reducing agent for the eco-friendly synthesis of AgNPs (referred to hereafter as TF-AgNPs) and AuNPs (referred to hereafter as TF-AuNPs). Surface plasmon resonance (SPR) of TF-AgNPs and TF-AuNPs was observed by acquiring UV-visible spectra. Microscopic tools, including field emission transmission electron microscopy (FE-TEM) and atomic force microscopy (AFM), were applied to characterize the size and shape. The colloidal stability of each nanoparticle solution was evaluated by acquiring UV-visible spectra. Measurements of hydrodynamic size and zeta potentials were conducted. To explore applications in future nanomedicine, the evaluation of antibacterial activity in terms of minimal inhibitory concentration (MIC) was conducted against both Gram-positive and Gram-negative bacteria. Cytotoxicity by a 3-(4,5-dimethylthiazol-2-yl)-2,5-diphenyltetrazolium bromide (MTT) assay was performed against a human gastric adenocarcinoma cell (AGS), a human colorectal adenocarcinoma cell (HT-29), and a human pancreas ductal adenocarcinoma cell (PANC-1) in order to see the possibilities of these nanoparticles as a chemotherapeutic agent in the treatment of cancers. A schematic presentation of the current report is depicted in Figure 1.

## 2. Materials and Methods

### 2.1. Materials

Sesquiterpenoids from the flower bud extract of *T. farfara* were obtained by counter-current chromatography according to a previous report [42]. We labeled this sample as ‘extract’ and used it for the synthesis. Silver nitrate and potassium gold(III) chloride were obtained from Sigma-Aldrich (St. Louis, MO, USA). Fetal bovine serum (FBS) was purchased from the GE Healthcare HyClone™ (Victoria, Australia). Cell culture reagents, including high-glucose Dulbecco’s modified Eagle’s medium (DMEM), trypsin-EDTA (0.5%, without phenol red), phosphate-buffered saline (PBS), and penicillin-streptomycin (10,000 U/mL), were obtained from Gibco (Thermo Fisher Scientific, Waltham, MA, USA).

### 2.2. Instruments

A Shimadzu UV-2600 UV-visible spectrophotometer was used to follow the reaction progress and assess the colloidal stability of nanoparticle solutions (Shimadzu Corporation, Kyoto, Japan). Spectra were acquired in the range of 300–800 nm using a quartz cuvette (1.5 mL). The crystallinity of nanoparticles was investigated using a high-resolution X-ray diffraction (HR-XRD) with a CuKα radiation source (λ = 0.154056 nm) (Ultima IV, Rigaku, Japan). Nanoparticle solutions were freeze-dried to obtain the X-ray diffraction pattern. To elucidate the crystalline structure, we used a library program that was installed in the XRD instrument. A JEM-2100F FE-TEM operating at 200 kV was used to acquire the size and shape information of the nanoparticles (JEOL, Tokyo, Japan). Nanoparticle solution was loaded onto a carbon-coated copper grid (carbon type-B, 300 mesh, Ted Pella, Redding, CA, USA) and allowed to dry at 37 °C oven for 24 h. A Dimension^®^ Icon^®^ operated in tapping mode was conducted to gain AFM images (Bruker Nano, Santa Barbara, CA, USA). The colloidal solution of nanoparticles was loaded onto a mica substrate (grade V-1, 25 mm by 25 mm perimeter, 0.15 mm thick, SPI Supplies Division of Structure Probe, Inc., West Chester, PA, USA) to acquire AFM images. Hydrodynamic size and zeta potentials were recorded on a NanoBrook 90Plus Zeta (Brookhaven Instruments Corporation, New York, USA).

### 2.3. Eco-Friendly Synthesis of TF-AgNPs and TF-AuNPs

In order to find the optimized reaction condition, each concentration of silver nitrate (for TF-AgNPs) or potassium gold(III) chloride (for TF-AuNPs), the extract and sodium hydroxide was varied together with the reaction time. The optimized reaction condition was tested on an 80 °C dry oven. One reaction variable was varied while the other variables were held constant. Three stock solutions were prepared for the synthesis in the following: silver nitrate (10 mM), the extract (0.1605% in 10% ethanol, w/v) and sodium hydroxide solution (100 mM). For the synthesis of TF-AgNPs, the extract (50 μL) was mixed with ethanol (100 μL), and the mixture was vortexed for several seconds. Sodium hydroxide (10 μL) and silver nitrate (50 μL) were added to this mixture, and the final volume was adjusted to 1 mL with deionized water. The final concentration of each in the reaction mixture was 0.008% (w/v) for the extract, 1 mM for sodium hydroxide, and 0.5 mM for silver nitrate. Then, the reaction mixture was vortexed for 10 s, and the incubation was performed on an 80 °C dry oven for 4 h or 24 h. The shape of the UV-visible spectra of TF-AgNPs was the same when the reaction time was either 4 h or 24 h. In the case of TF-AuNPs, the synthetic procedure was the same as TF-AgNPs, as mentioned above, in which potassium gold(III) chloride was used instead of silver nitrate. The incubation time was 2 h.

### 2.4. Colloidal Stability

The colloidal stability of the nanoparticle solution was assessed by two methods according to our previous report [43,44]: (i) stability under a salt, buffer, and cell culture medium and (ii) on-the-shelf stability for 20 days at ambient temperature at fixed time intervals as follows—day 2, 5, 10, 15, and 20 for TF-AgNPs, and day 1, 5, 12, and 20 for TF-AuNPs. For method (i), five different solutions were used to evaluate the colloidal stability of the nanoparticle solution: deionized water, PBS (pH 7.4), NaCl (1.8%, w/v), DMEM (phenol red-free medium), and a full medium. The full medium consisted of DMEM together with FBS (10%, w/v). In a 4 mL-glass vial, the mixture was prepared by mixing an equal volume (0.6 mL) of each and TF-AgNPs (0.6 mL). Then, the mixture was vortexed for 5 s and incubated for 30 min in a dry oven at 37 °C. After incubation, UV-visible spectra were acquired in the range of 300–800 nm, and the spectra were not elaborated. For method (ii), the on-the-shelf stability of both nanoparticle solutions was assessed for 20 days at ambient temperature. At fixed time intervals, UV-visible spectra were acquired in the range of 300–800 nm, and the spectra were not elaborated. Before acquiring UV-visible spectra, the nanoparticle solution was vortexed and sonicated.

### 2.5. Assessment of Antibacterial Activity

To assess the antibacterial activity of TF-AgNPs in terms of the MIC, two samples were prepared in aqueous ethanol (10%): (i) an extract (0.008%, w/v) and (ii) a TF-AgNPs (0.008%, w/v, based on extract concentration). The following four strains were used as quality controls: *Escherichia coli* ATCC 25922, *Enterococcus faecalis* ATCC 29212, *Pseudomonas aeruginosa* ATCC 27853, and *Staphylococcus aureus* ATCC 29213. Norfloxacin was selected as a standard. Twenty strains of Gram-positive and Gram-negative bacteria were tested for this purpose (Table 1). The MIC was determined in a 90 mm-dish according to our previous report [45]. The final concentration of each sample was diluted to the range of 1/2~1/1024 of the original sample. 

### 2.6. Cell Culture and Cytotoxicity

Cell culture and cytotoxicity was conducted according to our previous report [43,44]. Three cancer cell lines (AGS, HT-29 and PANC-1) were purchased from the Korean Cell Line Bank (Seoul, Republic of Korea). Cells were grown in high-glucose DMEM supplemented with penicillin (100 units/mL), streptomycin (100 μg/mL) and 10% FBS. Then, the cells were incubated at 37 °C (under 5% CO_2_) and preserved at approximately 80% confluence prior to trypsin/EDTA treatment. Each cancer cell line was seeded on 96-well plates with a density of 5.0 × 10^3^ cells per well. Then, the incubation was performed for 24 h in a 37 °C incubator under CO_2_ (5%). Five different concentrations of each nanoparticle solution (50.0, 25.0, 12.5, 6.25, and 3.12 μM Au or Ag) were treated on the cells [43,44], and the treated cells were incubated in a 37 °C incubator for an additional 24 h under CO_2_ (5%). The cells that were not treated were used as a control. Next, an MTT reagent (10 μL, 5% in deionized water) was added, and the incubation was conducted for an additional 3 h in a 37 °C incubator under CO_2_ (5%). A multi-detection microplate reader was used to record the absorbance at 570 nm (Synergy HT, Bio Tek Instruments, Winooski, VT, USA). All cytotoxicity experiments were performed in triplicates.

## 3. Results and Discussion

### 3.1. UV-visible Spectra

**Figure 2:**
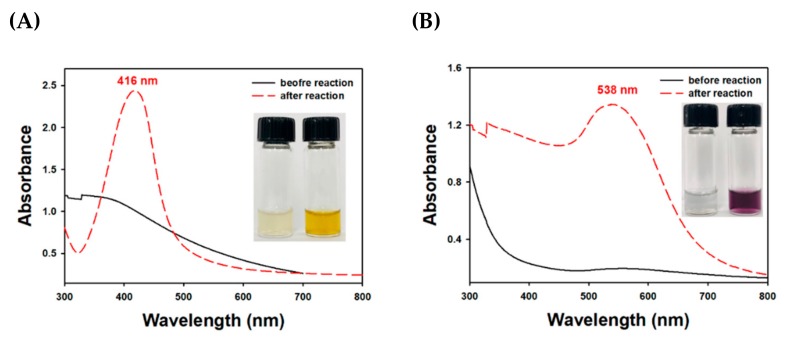
UV-visible spectra of (**A**) TF-AgNPs and (**B**) *T. farfara* gold nanoparticles (TF-AuNPs). The inset shows the digital photographs before oven incubation (left) and after oven incubation (right) in each inset.

Sesquiterpenoids are a C15 compound, and their biosynthetic pathway uses farnesyl pyrophosphate as a precursor. The chemical structures of three major sesquiterpenoids from *T. farfara* flower bud extract were reported in the previous report; tussilagone, 14-acetoxy-7β-(3′-ethyl *cis*-crotonoyloxy)-1α-(2′-methylburyryloxy)-notonipetranone, and 7β-(3′-ethyl *cis*-crotonoyloxy)-1α-(2′-methylburyryloxy)-3,14-dehydro-Z-notonipetranone [42]. These sesquiterpenoids were enriched by counter-current chromatography according to the previous report [42]. As shown in Figure 2, TF-AgNPs had a characteristic SPR band at 416 nm, suggesting that the synthesis was successful. Before the reaction, the color of the reaction mixture was close to colorless (Figure 2A, inset, left digital image) without distinct SPR. After the reaction, the solution color changed to yellow (Figure 2A, inset, right digital image). TF-AuNPs showed a characteristic SPR at 538 nm with a violet colored solution (Figure 2B, inset, right digital image).

### 3.2. HR-XRD Analysis

**Figure 3:**
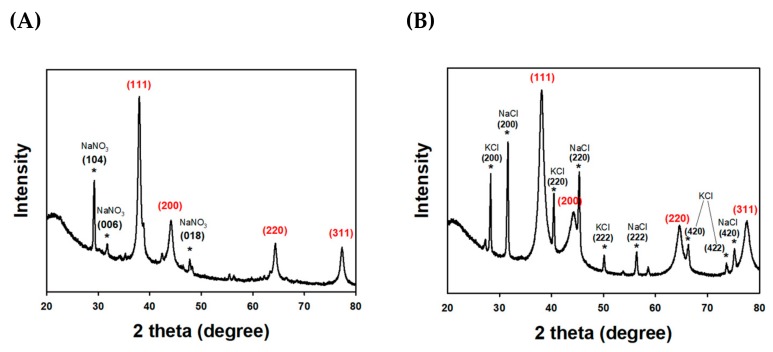
High resolution X-ray diffraction (HR-XRD) analysis of (**A**) TF-AgNPs and (**B**) TF-AuNPs. The red labeled peaks were from nanoparticles, whereas the asterisk (black labeled peaks) indicated the by-products from the reaction.

The crystalline characteristics of TF-AgNPs were evaluated by HR-XRD analysis, as shown in Figure 3A. Face-centered cubic XRD peaks, namely (111), (200), (220) and (311), emerged at 2θ values of 37.9°, 44.0°, 64.0°, and 76.9°, respectively. In the case of the TF-AuNPs, the four peaks at 38.1° for (111), 44.3° for (200), 64.5° for (220), and 77.5° for (311) indicated the face-centered cubic structure of TF-AuNPs. The XRD pattern of both nanoparticles was similar to previous reports [56,57]. The nanoparticle size was calculated by the Scherrer equation: D (size) = [0.94 × λ (X-ray wavelength)/W (the full width at half maximum in radians) × cos θ (the Bragg diffraction angle)]. The size of TF-AgNPs was calculated as 15.8 nm from the (111) peak, 10.3 nm from the (200) peak, 19.9 nm from the (220) peak, and 16.4 nm from the (311) peak. From this result, the average size of TF-AgNPs was estimated to be 15.6 nm. The other three peaks with asterisks were observed at 29.2°, 31.6°, and 47.6° for the (104), (006), and (018) planes, respectively. These three peaks were from NaNO_3_ impurity, which was a by-product of the reaction. The HR-XRD analysis results of TF-AuNPs are shown in Figure 3B. The size of TF-AuNPs was estimated from the Scherrer equation as 8.0 nm from (111), 4.6 nm from (200), 7.4 nm from (220), and 7.8 nm from (311). From this result, the average size of the TF-AgNPs was estimated to be 7.0 nm. The contaminants were identified as KCl and NaCl, which were by-products of the reaction. The KCl peaks were observed at 28.3°, 40.5°, 50.2°, 66.4°, and 73.7° corresponding to (200), (220), (222), (420), and (422), respectively. The NaCl peaks were observed at 31.6°, 45.3°, 56.3°, and 75.1° for the (200), (220), (222), and (420) planes, respectively.

### 3.3. FE-TEM and AFM Images

**Figure 4:**
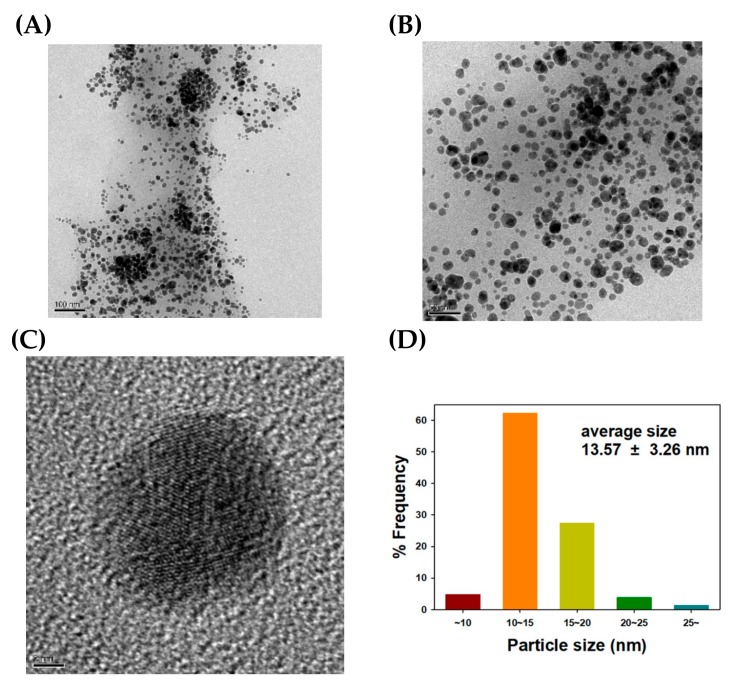
Field emission transmission electron microscopy (FE-TEM) images and a size histogram of TF-AgNPs. The scale bar represents (**A**) 100 nm, (**B**) 50 nm, and (**C**) 2 nm. (**D**) A size histogram was determined from the measurement of 207 discrete nanoparticles in FE-TEM images using the Image J program.

FE-TEM images of the TF-AgNPs are displayed in Figure 4. Spherical particles were mostly synthesized, and the lattice fringes are clearly seen in Figure 4C. The presence of lattice fringes confirmed the crystalline nature of the TF-AgNPs, which is consistent with the HR-XRD analysis in the previous section. From 207 discrete nanoparticles, the average size was measured to be 13.57 ± 3.26 nm with the aid of the Image J program. The size histogram is provided in Figure 4D. The most frequent size was determined to be 10 nm to 15 nm (62% frequency). 

**Figure 5:**
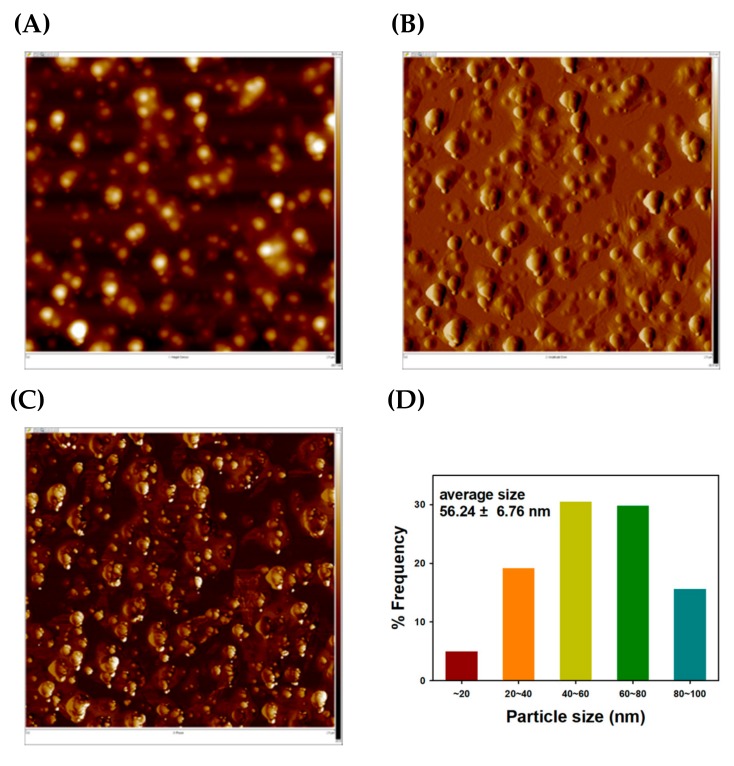
Atomic force microscopy (AFM) images and a size histogram of TF-AgNPs. (**A**) height sensor image (2.5 μm × 2.5 μm), (**B**) amplitude error image (2.5 μm × 2.5 μm), (**C**) phase image (2.5 μm × 2.5 μm), and (**D**) a size histogram.

Next, AFM images were acquired and are shown in Figure 5. Similar to FE-TEM images, mostly spherical-shaped TF-AgNPs were observed in the images. In the height sensor image in Figure 5A, the bright-colored and spherical-shaped particles are TF-AgNPs. Both an amplitude error image (Figure 5B) and a phase image (Figure 5C) clearly show these nanoparticles. The average size was also measured from the AFM images, and the size histogram is shown in Figure 5D. The size was 56.24 ± 6.76 nm from 141 discrete nanoparticles in the image, which was larger than that measured from FE-TEM images. This is possibly due to the cold welding phenomenon of AgNPs on a mica substrate [46].

**Figure 6:**
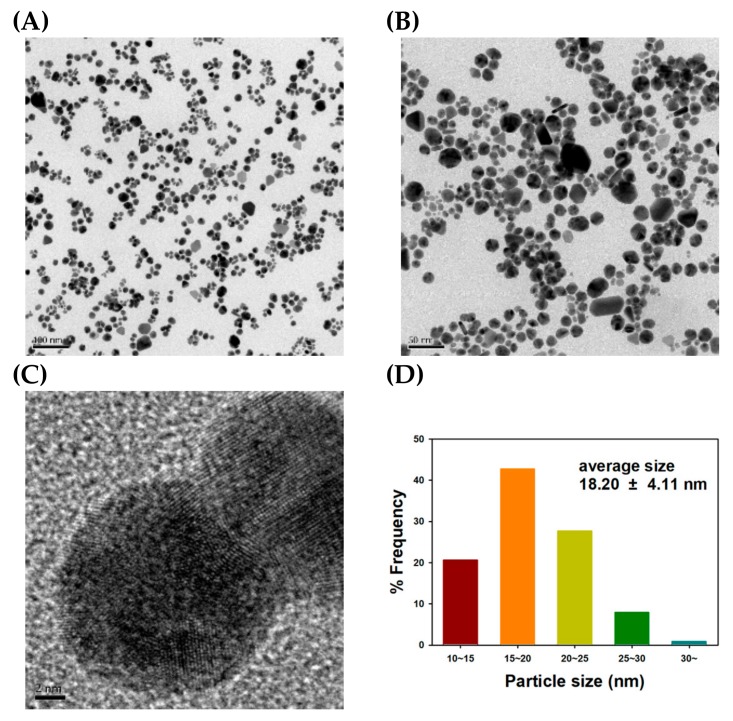
FE-TEM images and a size histogram of TF-AuNPs. The scale bar represents (**A**) 100 nm, (**B**) 50 nm and (**C**) 2 nm. (**D**) A size histogram was determined from the measurement of 213 discreet nanoparticles in FE-TEM images using the Image J program.

**Figure 7:**
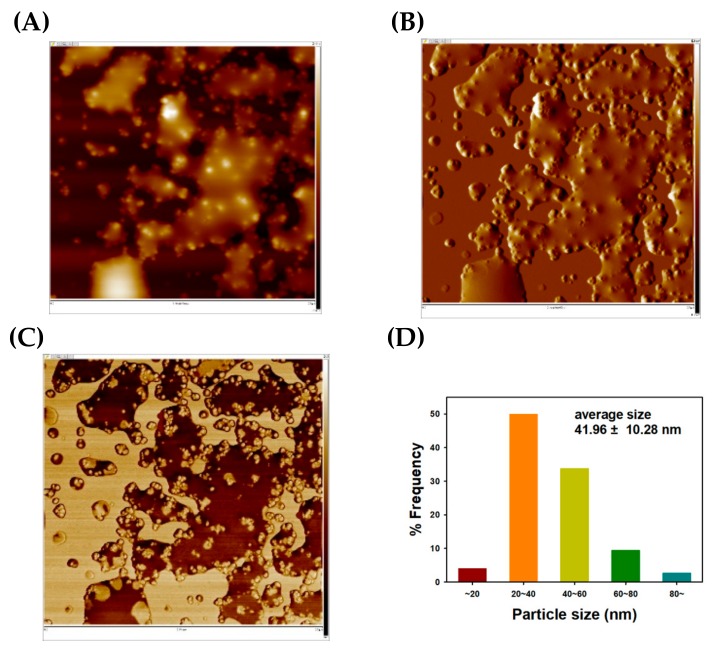
AFM images and a size histogram of TF-AuNPs. (**A**) height sensor image (2.5 μm × 2.5 μm), (**B**) amplitude error image (2.5 μm × 2.5 μm), (**C**) phase image (2.5 μm × 2.5 μm), and (**D**) a size histogram.

As shown in Figure 6, the spherical-shaped TF-AuNPs can be observed together with clear lattice fringes in Figure 6C. The observation of the lattice fringes also confirms the crystalline nature of the TF-AuNPs, which corroborates the HR-XRD analysis in the previous section. From 213 discrete nanoparticles, the average size was estimated as 18.20 ± 4.11 nm (Figure 6D). The most frequent size observed was 15 nm to 20 nm (43% frequency). AFM images are shown in Figure 7. 

The liquid-like phase in AFM images was observed in Figure 7A–C. This was due to existence of reducing agents in nanoparticle solution. Similar to TF-AgNPs, bright-colored and spherically shaped TF-AuNPs can be observed in the height sensor image (Figure 7A). The average size from AFM images was 41.96 ± 10.28 nm from 74 discrete TF-AuNPs (Figure 7D). The size discrepancy between FE-TEM and AFM images was due to the cold welding phenomenon of AuNPs on the mica substrate [47].

### 3.4. Hydrodynamic Size and Zeta Potential Measurements

Along with the size measurement from the FE-TEM images, the hydrodynamic size was also measured by dynamic light scattering. For TF-AgNPs, the hydrodynamic size was determined to be 202 nm with a polydispersity index of 0.249. A large negative zeta potential was observed to be −36.9 mV, suggesting that the TF-AgNP colloidal solution was quite stable. In the case of TF-AuNPs, the hydrodynamic size, polydispersity index, and zeta potential were 92.9 nm, 0.265, and −14.5 mV, respectively. Hydrodynamic size was larger than that measured from FE-TEM images in the previous section. This is possibly because sesquiterpenoids in the extract were bound to the surface of nanoparticles, contributing to the increase in size in a solution state. The same phenomenon was reported by Rolim and co-workers as follows. They synthesized AgNPs with a green tea extract via a green strategy. “The hydrodynamic size of the nanoparticles was found to be higher in comparison to the average size observed at solid state. The presence of extra hydrate layers, along with ions or molecules attached to the nanoparticle surface in an aqueous environment, was responsible for the higher hydrodynamic sizes [48]”. The same results were also obtained in our previous reports [44,49,50].

### 3.5. Assessment of Colloidal Stability

**Figure 8:**
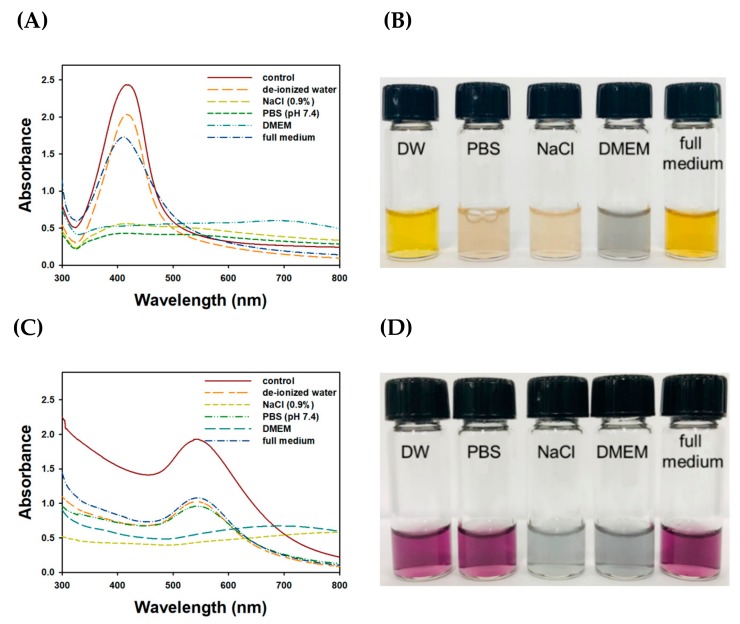
Assessment of colloidal stability under five different solutions: deionized water, NaCl (0.9%), phosphate-buffered saline (PBS) (pH 7.4), Dulbecco’s modified Eagle’s medium (DMEM), and full medium. (**A**) UV-visible spectra of TF-AgNPs, (**B**) digital images of colloidal TF-AgNPs in (**A**) spectra, (**C**) UV-visible spectra of TF-AuNPs and (**D**) digital images of colloidal TF-AuNPs in (**C**) spectra.

**Figure 9:**
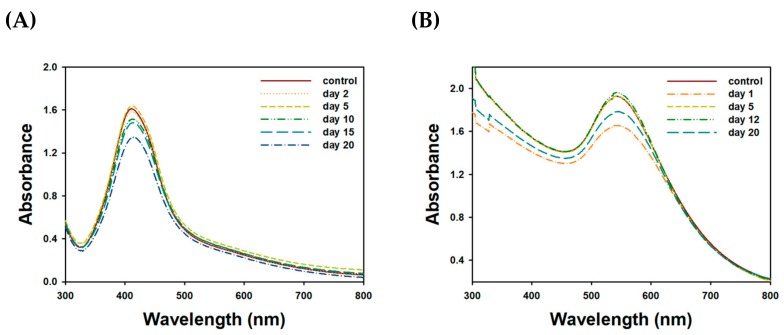
Assessment of on-the-shelf colloidal stability for 20 days at ambient temperature. UV-visible spectra of (**A**) TF-AgNPs and (**B**) TF-AuNPs.

Firstly, the results of colloidal stability under five different solutions are shown in the UV-visible spectra (Figure 8). When compared to control spectra, the maximum UV absorbance decreased for all tested solutions. SPR of TF-AgNPs in each solution was as follows; control (416 nm), de-ionized water (416 nm), full medium (411 nm). SPR observation in NaCl (0.9%), PBS (pH 7.4), and DMEM solution was not successful. These results indicate that the colloidal stability of the TF-AgNPs was maintained best in deionized water, followed by a full medium (Figure 8A). However, with the other solutions (i.e., PBS (pH 7.4), NaCl (0.9%), and DMEM) the characteristic shape of the UV-visible spectra totally disappeared, implying that the colloidal stability in these solutions was very low. The results from TF-AuNPs are shown in Figure 8C. The SPR of TF-AuNPs in each solution was as follows: control (542 nm), de-ionized water (542 nm), PBS (pH 7.4) (544 nm), and full medium (544 nm). SPR observation in the NaCl (0.9%) and DMEM solutions was not successful. Three solutions (i.e., full medium, deionized water, and PBS) slightly decreased UV absorbance to the same degree. However, the colloidal stability in DMEM and NaCl (0.9%) was very low. It is important to note that the shape of the UV-visible spectra was reasonably retained in a full medium in both nanoparticles. Therefore, the colloidal stability was retained during the cytotoxicity experiment using a full medium. Secondly, on-the-shelf colloidal stability was assessed at ambient temperature for 20 days, and the result is shown in Figure 9. A slight hypochromic shift was observed in both nanoparticles. An absorbance decrease (hypochromic shift) of TF-AuNPs at 542 nm and TF-AgNPs at 412 nm was 7.5% and 16.6%, respectively. The shape of the SPR was reasonably retained. Furthermore, no aggregation of nanoparticles was detected. Thus, the sesquiterpenoids in the extract possibly played a role as a stabilizing agent. The colloidal stability result was comparable to the previous report, in which AuNPs were capped with chitosan [44].

### 3.6. Antibacterial Activity of TF-AgNPs

The assessment of antibacterial activity is shown in Table 1 with twenty tested strains and four quality control strains. MIC values are expressed as the extract concentration. When both the extract and TF-AgNPs are compared, TF-AgNPs show better activity (two- to four-fold enhancement) than the extract alone among the tested strains. This result suggests that AgNPs reinforced the antibacterial activity of the extract. The highest antibacterial activity (that means the lowest MIC value, 10 μg/mL) of TF-AgNPs was observed in the following strains: *Enterococcus faecium* (CCARM 5262, susceptible), *Enterococcus faecium* (CCARM 5024, vancomycin-resistant Enterococci, Van-A), *Escherichia coli* (CCARM 0230, 0236, 0237 and 0238), *Pseudomonas aeruginosa* (CCARM 0225), *Salmonella typhimurium* (CCARM 0240), *Klebsiella oxytoca* (CCARM 0248), and *Enterobacter cloacae* (CCARM 0252 and 0253). Among these strains, *Enterococcus faecium* is a Gram-positive bacterium, and the rest are Gram-negative bacteria. Gram-negative bacteria are more susceptible than Gram-positive bacteria to the treatment of AgNPs [51,52,53,54,55]. Most surprisingly, TF-AgNPs showed the highest activity against *Enterococcus faecium* (CCARM 5024), which is a Van-A type vancomycin-resistant Enterococci (VRE). VRE are bacterial strains that are resistant to vancomycin treatment. Specifically, Van-A type VRE are resistant to the treatment by both vancomycin and teicoplanin. The MIC value of TF-AgNPs against this strain was 10 μg/mL, and the MIC of a standard antibiotic, norfloxacin, was 8 μg/mL. Therefore, TF-AgNPs may be a candidate for the treatment of VRE as an antibacterial agent. In our previous report, stabilizers profoundly affected antibacterial activity of AgNPs which was green-synthesized with *Artemisia capillaris* extract [56]. Two stabilizers with different charges were tested: cetyltrimethylammonium bromide (CTAB, positive charged) and sodium dodecyl sulfate (SDS, negative charged). The CTAB-stabilized AgNPs exhibited the highest antibacterial activity against methicillin-resistant *Staphylococcus aureus* (MRSA) among the extract, AgNPs prepared in the absence of stabilizers, and SDS-stabilized AgNPs [56]. The AgNPs green-synthesized with *Caesalpinia sappan* extract also exerted potent antibacterial activity against MRSA when CTAB was used as a stabilizer [57]. The positive charge of the CTAB-stabilized AgNPs are likely to enhance their affinity to the negatively charged bacterial cell wall. Along with the charges, the shape also affected the antibacterial activity of AgNPs. Triangular AgNPs are more effective against *Escherichia coli* than spherical and rod-shaped AgNPs [14]. Therefore, overall charge and shape of AgNPs should be considered when synthesizing AgNPs using plant extracts for antibacterial applications.

### 3.7. Cytotoxicity Assessment of TF-AgNPs and TF-AuNPs

**Figure 10:**
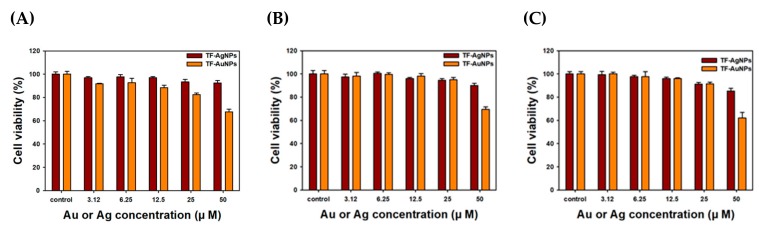
3-(4,5-dimethylthiazol-2-yl)-2,5-diphenyltetrazolium bromide (MTT) assay on (**A**) human gastric adenocarcinoma cell (AGS), (**B**) human colorectal adenocarcinoma cell (HT-29) and (**C**) human pancreas ductal adenocarcinoma cell (PANC-1) cells.

Important features of AuNPs and AgNPs include their participation in cancer diagnostics and therapeutics. Recently, nanoparticles have emerged as a promising tool offering theranostic applications [20,58,59,60,61]. Thus, we selected four cancer cell lines to pursue possibilities for the development of anticancer agents or vehicles/carriers for biologically active molecules. The results of the MTT assay are displayed in Figure 10. At the highest concentration of 50.0 μM Au or Ag, TF-AuNPs were more cytotoxic than TF-AgNPs in all three cell lines. As shown in Table 2, the IC_50_ values of PANC-1 cells were the lowest: 166.1 μM Ag for TF-AgNPs and 71.2 μM Au for TF-AuNPs. The results suggested that both TF-AgNPs and TF-AuNPs can be used as drug delivery vehicles or carriers for anticancer agents. 

## 4. Conclusions

Sesquiterpenoids from *T. farfara* flower bud extract were effectively used as a reducing agent for the green synthesis of TF-AgNPs and TF-AuNPs. The current green strategy was straightforward and simple; thus, it can be applicable to extracts of natural products. UV-visible spectra, X-ray diffraction patterns, FE-TEM, and AFM images confirmed that TF-AgNPs and TF-AuNPs were spherically shaped and possessed a face-centered cubic structure. The MIC method revealed that, among the bacteria strains considered in this study, TF-AgNPs had the highest antibacterial activity against vancomycin-resistant Enterococci Van-A type *Enterococcus faecium*. This result suggested that TF-AgNPs have the potential to be an effective nano-platform for a novel antibacterial agent. Cytotoxicity on the three cancer cell lines, including AGS, HT-29, and PANC-1, revealed that TF-AuNPs were more cytotoxic than TF-AgNPs. Among the three tested cancer cell lines, the highest cytotoxicity was observed in PANC-1 cells. Therefore, doxorubicin will be loaded on the PEGylated TF-AuNPs, and their cytotoxicity will be evaluated in our future work for nanomedicine applications.

## Figures and Tables

**Table 1:** Antibacterial activity of the extract and *T. farfara* silver nanoparticles (TF-AgNPs) measured by the minimal inhibitory concentration (MIC) method.

No.	Strain	MIC (μg/mL)		
		Extract	TF-AgNPs	Nor ^a^
1	*Enterococcus faecalis* CCARM 5171 (Susceptible)	>40	40	4
2	*Enterococcus faecium* CCARM 5262 (Susceptible)	>40	10	1
3	*Enterococcus faecalis* CCARM 5025 (Vancomycin-Resistant Enterococci, Van-B)	>40	>40	2
4	*Enterococcus faecium* CCARM 5024 (Vancomycin-Resistant Enterococci, Van-A)	>40	10	8
5	*Staphylococcus aureus* CCARM 0205 (Susceptible)	40	20	1
6	*Staphylococcus aureus* CCARM 3855 (Susceptible)	>40	>40	1
7	*Staphylococcus aureus* CCARM 3089 (Multiple Drug Resistant)	>40	40	128
8	*Escherichia coli* CCARM 0230	>40	10	≤0.03
9	*Escherichia coli* CCARM 0235	20	20	0.06
10	*Escherichia coli* CCARM 0236	>40	10	0.06
11	*Escherichia coli* CCARM 0237	>40	10	1
12	*Escherichia coli* CCARM 0238	>40	10	0.25
13	*Pseudomonas aeruginosa* CCARM 0219	>40	20	1
14	*Pseudomonas aeruginosa* CCARM 0223	>40	40	0.5
15	*Pseudomonas aeruginosa* CCARM 0225	20	10	0.06
16	*Salmonella typhimurium* CCARM 0240	>40	10	0.06
17	*Klebsiella oxytoca* CCARM 0248	>40	10	≤0.03
18	*Klebsiella aerogenes* CCARM 0249	>40	20	0.12
19	*Enterobacter cloacae* CCARM 0252	>40	10	0.06
20	*Enterobacter cloacae* CCARM 0253	>40	10	≤0.03
21	*Escherichia coli* ATCC 25922 (QC strain)	>40	10	0.06
22	*Enterococcus faecalis* ATCC 29212 (QC strain)	>40	>40	2
23	*Pseudomonas aeruginosa* ATCC 27853 (QC strain)	>40	10	1
24	*Staphylococcus aureus* ATCC 29213 (QC strain)	>40	40	1

^a^: norfloxacin, a standard.

**Table 2:** MIC of TF-AgNPs and TF-AuNPs on three cancer cell lines.

MIC (μM)	Cancer Cell Lines		
	AGS	HT-29	PANC-1
TF-AgNPs	338.0	275.3	166.1
TF-AuNPs	77.9	87.0	71.2
